# Devastating Complications of Metal Strut Migration Following Pectus Excavatum Repair

**DOI:** 10.1055/s-0039-1692195

**Published:** 2019-07-12

**Authors:** Mila Stajevic, Milorad Bijelovic, Jovan Kosutic, Radoje Simic

**Affiliations:** 1 School of Medicine, University of Belgrade, Belgrade, Serbia; 2 Department of Pediatric Cardiothoracic Surgery, Institute for Mother and Child Health Care “Dr Vukan Cupic”, Belgrade, Serbia; 3 Faculty of Medicine, University of Novi Sad, Novi Sad, Serbia; 4 Clinic for Thoracic Surgery, Institute for Pulmonary Diseases of Vojvodina, Sremska Kamenica, Serbia; 5 Department of Pediatric Cardiology, Institute for Mother and Child Health Care “Dr Vukan Cupic”, Belgrade, Serbia; 6 Department for Plastic and Reconstructive Surgery and Burns, Institute for Mother and Child Health Care “Dr Vukan Cupic”, Belgrade, Serbia

**Keywords:** pectus excavatum, the Ravitch procedure, strut migration, complications

## Abstract

The modified Ravitch technique with metal struts and the Nuss operation have been the dominant operative techniques for treatment of pectus excavatum in the previous decades. We present devastating postoperative complications of a 16-year-old boy after the modified Ravitch procedure for a severe deformity utilizing two metal bars. Four months following surgery, one strut was removed after the displacement noted on a regular postoperative examination. Ten days after the strut removal, the patient complained of lower limb pain but the sensations were attributed to physical inactivity. Two months later, after pain intensification, the boy was diagnosed with bilateral arterial and venous lower limb thromboses and subsequently, the migration of the remaining metal strut intracardially with the free end in the left ventricular cavity embedded in massive thrombi. An urgent cardiac procedure was performed and the bar removed. Postoperatively, the boy made a full cardiac recovery but with severe neurological complications and subsequent death. Migration of metal struts is a rare complication and, except in our case, had been dealt with successfully. This case should emphasize more attention to the postoperative follow-up management of such patients.

## Introduction

The Nuss procedure and the modified Ravitch technique are the most commonly performed operations for pediatric anterior chest wall deformities. 1 The modified Ravitch procedure includes lower sternum exposure, removal of abnormal cartilages, and fixation of the sternum with a stainless steel bar which is left in place for at least a year and is removed afterwards. The metal strut displacement after the modified Ravitch procedure is extremely rare and can have intrathoracic or intraabdominal propagation. 2 3 4 5 6 7 There are less than 20 reports in the literature of patients whose postoperative course had been complicated by migration of the metal strut into the pericardium, right atrium, left and right ventricle, the abdomen, and left upper bronchus. 8 Our patient had been operated with two stainless steel 30 cm struts with end perforations; the technique being chosen due to the severity and asymmetry of the pectus excavatum (PE).

## Case Report

A 16-year-old boy with a severe deformity underwent a modified Ravitch procedure in an institution. Two metal struts were inserted to stabilize the deformity and were sewn to the ribs with heavy No. 2 multifilament nonabsorbable braided sutures. The position of the bars was such that the tension had been larger on the inferior strut due to the asymmetry of the PE. The position of the metal bars had been verified postoperatively by anteroposterior and lateral chest X-ray only. The displacement of one strut was noted after 4 months. The inferior strut migrated approximately 1cm rightward and threatened to perforate the skin. The mechanism of migration was straightening of the strut due to the severity of the intrathoracic deformity, its strong pulling force and consequent displacement. The dislocated bar was surgically removed and the other strut left in place. The postoperative course was uneventful and the position of the remaining bar checked only by radiography. At the time of initial surgery, the laboratory results were consistent with the findings of a healthy young adolescent.

The patient complained of limb pain 10 days post strut removal. With pain intensification, he was examined by a vascular surgeon about 2 months after the second intervention. Bilateral distal arterial and venous leg thromboses were diagnosed by the color Doppler. The transthoracic heart ultrasound showed the remaining metal strut perforating the anterior right ventricular (RV) wall, the intraventricular septum, ending its course in the left ventricular (LV) cavity with massive intracardiac biventricular thrombosis with no pericardial effusion. He was immediately transferred to a pediatric cardiac surgery center.

**Fig. 1:**
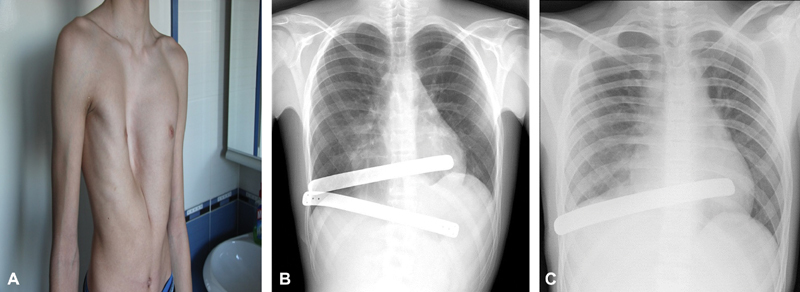
( **A** ) Preoperative severe pectus excavatum; ( **B** ) displacement of the inferior metal strut; ( **C** ) the postoperative position of the remaining strut.

On admission, the boy was neurologically intact, with no clinical signs of venous obstruction. The femoral arterial pulses were vaguely present, the popliteal were absent. An urgent multi detector computer tomography (MDCT) was performed. The brain scan was normal. The superior vena cava (SVC) pathway was clear, but the inferior vena cava (IVC) system was partially occluded with two long plaques below the cavoatrial junction ( Fig. 1 ).

**Fig. 2:**
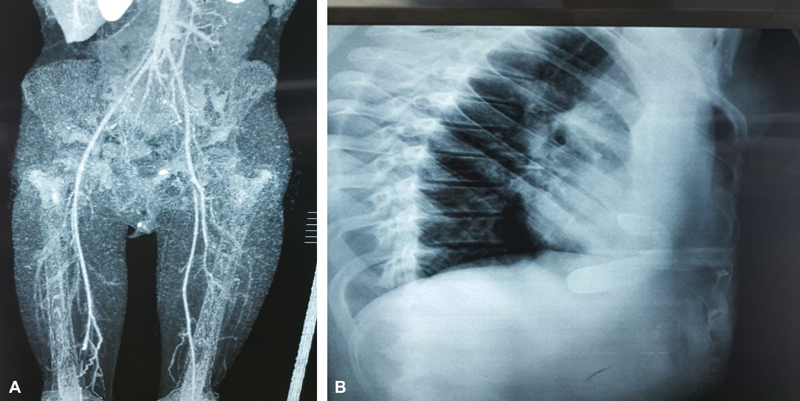
( **A** ) Lower limb angiography showing partial occlusion of both femoral arteries and absent circulation in the popliteal arteries; ( **B** ) the lateral X-ray of the remaining strut.

The portal venous system was unobstructed, there was a partial occlusion of the left femoral vein. The arterial circulation was severely damaged, partial occlusion of both femoral arteries with no circulation in the popliteal and posterior tibial arteries. An indication for urgent cardiac operation and strut removal was quoted to the parents with high intraoperative risks ( Fig. 2 ).

**Fig. 3:**
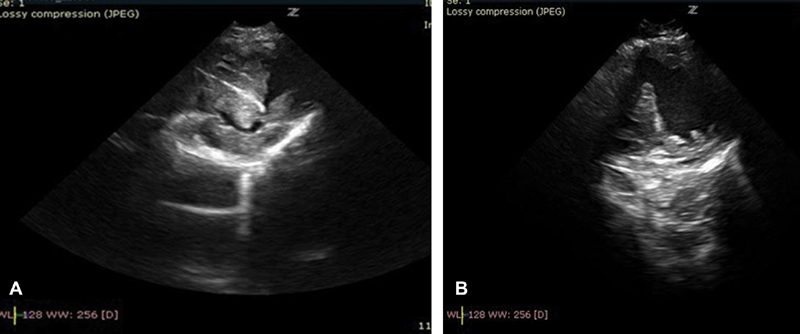
( **A** ) Intracardial metal strut ending its course in the left ventricle “muffed” by thrombus; ( **B** ) iatrogenic ventricular septal defect after strut and thrombus removal.

**Fig. 4:**
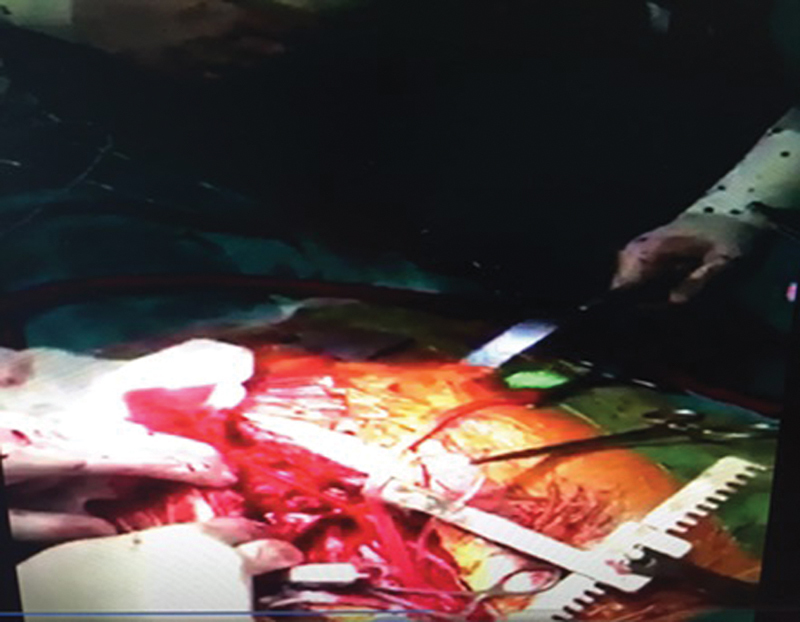
Intraoperative moment of metal strut removal.

The operation was performed on cardio pulmonary bypass (CPB) in deep hypothermia and neck cannulation. A transoesophageal echocardiogram (TOE) was performed during the surgery. The sternum was severely adherent to the pericardium with the lower left sided ribs, completely detached from the sternum. After the cardiac arrest, the strut was removed without resistance ( Figs. 3 and 4 ).

**Fig. 5:**
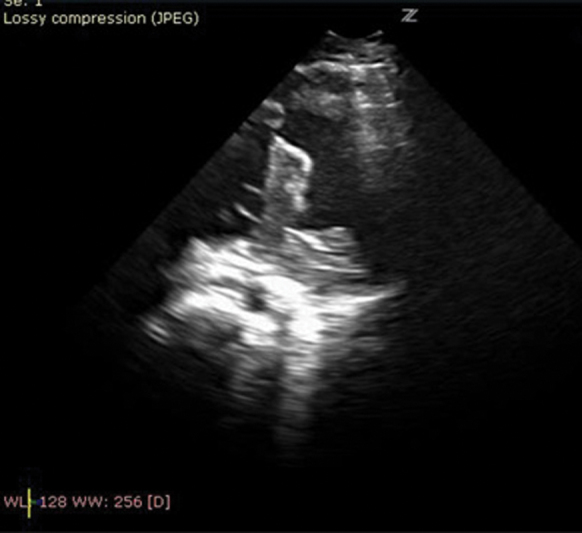
VSD closed with a PTFE patch with repair of the RV wall. PTFE, polytetrafluoroethylene; RV, right ventricular; VSD, ventricular septal defect.

The cardiac apex was macerated, the enterance hole on the parietal wall of the RV was “muffed” with thrombi and fibrin. The RV clots were removed through the undamaged tricuspid valve. The large iatrogenic ventricular septal defect (VSD) involved the cardiac apex with the surrounding muscular septum. Left-sided massive thrombi and fibrin detritus were evacuated transeptally and via the VSD. The mitral valve (MV) was intact. The VSD was closed with a 0.6 mm polytetrafluoroethylene (PTFE) patch and the cardiac apex reconstructed with direct pledgeted sutures ( Fig. 5 ).

The postoperative TOE showed poor myocardial function with severe dyskinesia of the ventricular septum but no residual masses. He was resuscitated for 30 minutes postoperatively for refractive ventricular fibrillations in spite of aggressive antiarrhythmic therapy and pacemaker overdrive. The patient was in junctional ectopic tachycardia (JET), required high-inotropic support, but converted into sinus rhythm within 12 hours. Control MDCT showed ischemic brain lesions with no bleeding. The laboratory tests did not imply of an underlying coagulopathy. The improved cardiac status allowed extubation within a week but due to further neurological deterioriation with frequent generalized convulsions, he was discharged severely impaired and transferred to his local hospital. Migration of metal struts is a rare complication and, except in our case, had been dealt with successfully.

## Discussion

The mechanism of migration has not been clearly identified. It should be differentiated from strut dislocation when displacement occurs without organ injury. In our case, the strut migration into the heart followed the removal of a dislocated metal strut less than 6 months after the PE repair which is one of the earliest found in the literature (6 months to 37 years). 6 8 There are no valid comparisons of the modified Ravitch technique strut migrations versus the Nuss bar migrations. The exact mechanism of this painless heart perforation is not known. The postoperative position of the remaining strut, in our case, was checked by anterioroposterior and lateral X-rays and was found to be sufficient and adequate. Our presumption is that, in our case, the left-sided sternal joints of the abnormal and fused ribs detached shortly after the first strut removal. The ribs had stabbed the myocardium but did not protrude into the ventricle. The loss of anchor of the single metal strut and the severity and asymmetry of the PE combined with the increased pressure, allowed the respiratory chest movements to slowly and gradually impress the strut into the heart. Therefore, we conclude that the second strut migration is the consequence of the first strut removal.

We would advocate postoperative X-ray screenings at 1, 3, 6, and 12 months postsurgery. A cardiac/Doppler ultrasound examination should be performed before the hospital discharge in cases of cardiovascular symptoms or eventful postoperative courses. It is questionable that should both bars be removed if one would get dislocated and the other one had seemed to remain in place. We feel that the decision should be individually made depending on the patient's condition, experience of the surgeon and the possibilities of the medical center. Considering that the metal struts can generate pressure on the myocardium, antiaggregational therapy can also be debated as a possible postoperative standard in cases where there is evidence of clinical symptoms rather than only aesthetic deformity.

The prevention of metal strut migrations can be prevented with longer devices which can include fixage of more than one rib.

## Conclusion

The modified Ravitch procedure is predominantly an esthetic operation performed in children with marked PE deformity and is considered safe and an alternative to the Nuss procedure. About 20 cases in the literature have been described where the metal stabilizators have migrated and caused different organ injuries. Our case with a fatal outcome raises awareness for the follow-up of these patients and immediate action, especially in the situation of strut dislocations or migrations.

## Notes

New Insights and the Importance for the Pediatric Surgeon This is a rare but fatal complication of an aesthetic operation in the pediatric age group. Very few reports have been made on this subject. We feel that we need to point the possibility of these complications.
